# New Modes of Preparing Objects for the Microscope

**Published:** 1869-02

**Authors:** 


New Modes of Preparing Objects for the Microscope. In Mr. Lockhart Clarke's essay on the Intimate Structure of the Brain, contained in the Philosophical Transactions for 1868, we read that Gerlach now hardens the cord in a solution of bichromate of ammonia of the strength of from one to two per cent. The sections are then put into a solution of one part of chloride of gold and potassium to 10,000 parts of water, slightly acidulated either with vinegar or hydrochloric acid, for ten or twelve hours; when the white substance becomes of a pale lilac, while the gray substance is scarcely colored. They are then placed in a mixture of one part of hydrochloric acid to 2,000 or 3,000 parts of water for a few minutes. After this, the sections are steeped for about ten minutes in a mixture of one part of hydrochloric acid to 1,000 parts of 60 per cent. alcohol, and then for some minutes in absolute alcohol; after which they are made transparent by means of creosote, and put up in Canada balsam. Mr. Clarke adds: "This is rather a complicated process." A much simpler method has been proposed by M. Rauvier in the last number of Brown-Sequard's *Archives of Physiology*, which consists in the employment of picric or carbazotic acid. This acid is only moderately soluble in water, and a saturated solution may therefore be employed. It possesses the further advantage of being very cheap. It is admirably adapted for all tissues containing much blood, and therefore for specimens of liver, lung, etc. It appears to act by effecting coagulation of the albuminous substances, though, unlike alcohol and chromic acid, it does not occasion any fusion of the constituents of the tissue. The red globules retain their form and characters extremely well. The portion of tissue required to be examined should be plunged into the solution, and after the lapse of twenty-four hours, it will be found to have acquired sufficient firmness to permit of very fine sections being made with a razor. The saving of time by this method, as compared with the chromic acid, is immense. The preparations will take color from carminate of ammonia, and may be prepared in glycerine.—*Lancet.*

